# Inspiratory muscle training facilitates liberation from mechanical ventilation in subacute critically ill patients—a randomized controlled trial

**DOI:** 10.3389/fmed.2024.1503678

**Published:** 2025-01-29

**Authors:** Shu-Jane Wang, Tien-Pei Fang, Daniel D. Rowley, Nan-Wei Liu, Jui-O Chen, Jui-Fang Liu, Hui-Ling Lin

**Affiliations:** ^1^Department of Respiratory Therapy, Zuoying Armed Forces General Hospital, Kaohsiung, Taiwan; ^2^Department of Respiratory Therapy, Chiayi Chang Gung Memorial Hospital, Chiayi, Taiwan; ^3^Department of Respiratory Care, Chang Gung University of Science and Technology, Chiayi, Taiwan; ^4^Respiratory Therapy Services, University of Virginia Medical Center, Charlottesville, VA, United States; ^5^Department of English, National Chengchi University, Taipei, Taiwan; ^6^Department of Nursing, Tajen University, Pingtung, Taiwan; ^7^Chronic Diseases and Health Promotion Research Center, Chang Gung University of Science and Technology, Chiayi, Taiwan; ^8^Department of Respiratory Therapy, Chang Gung University, Taoyuan, Taiwan

**Keywords:** inspiratory muscle training, subacute critical ill, mechanical ventilation support, rapid shallow breathing index, respiratory muscle strength, creatinine

## Abstract

**Background:**

Patients undergoing mechanical ventilation often develop rapid diaphragmatic atrophy, respiratory muscle weakness, and dysfunction, which are associated with prolonged duration of ventilation. This study aimed to evaluate whether Inspiratory Muscle Training (IMT) facilitates weaning from mechanical ventilation and enhances muscle strength in critically ill, subacute adult patients, while examining the relationship between IMT and relevant clinical laboratory values.

**Methods:**

In this randomized clinical trial, patients admitted to the intensive care unit requiring mechanical ventilation for more than 2 days, with stable hemodynamics and resolved acute conditions, were enrolled. Participants were randomly assigned to the IMT or no-IMT group. The IMT group received training twice daily, 5 days a week, for three consecutive weeks. The primary outcome was ventilator duration. The primary outcome measure was the number of days until liberation from mechanical ventilation. The secondary outcomes of interest were respiratory muscle strength and biomarker levels.

**Results:**

Thirty-three subjects (17 in the IMT group, 16 in the no-IMT group) were included in the final analysis. The IMT group had significantly shorter ventilator days (12.6 ± 5.2 vs. 18.1 ± 8.8, *p* = 0.04). IMT intervention significantly reduced rapid shallow breathing index and improved respiratory muscle strength, with greater maximum inspiratory pressure (*p* < 0.01), maximum expiratory pressure (*p* = 0.03), and peak expiratory flow (*p* = 0.01). A moderate positive correlation was observed between IMT and increased creatinine levels (rs = 0.54, *p* = 0.01), whereas the no-IMT group showed a reduction.

**Conclusion:**

IMT significantly shortened ventilator duration and improved respiratory muscle strength. A moderate correlation between increased creatinine levels and respiratory muscle strength was observed, suggesting that creatinine may be a potential biomarker for muscle recovery during IMT.

**Clinical trial registration:**

This study was registered at ClinicalTrials.gov (NCT06611683).

## 1 Introduction

Acute respiratory failure is the primary reason that mechanical ventilation is aimed at maintaining sufficient ventilation to support life. Patients on mechanical ventilation often experience rapid diaphragm atrophy on the second day, resulting in diaphragm myofiber changes, respiratory muscle weakness, dysfunction, and impaired physical function (1–3). These changes can cause severe complications such as frailty and infections, leading to an increased duration of mechanical ventilation, extended intensive care unit (ICU) stay, and higher 1-year mortality (4).

Clinical studies have explored enhancing diaphragm and respiratory muscle strength and endurance through inspiratory muscle, expiratory muscle, and combined respiratory muscle training (5, 6). Training methods include flow-resistive, pressure threshold, and resistance training using electronic devices (7, 8). These strategies enhance respiratory muscle strength and endurance, promoting muscle fiber overload through increased training intensity, duration, and frequency (9, 10). Clinical practice guidelines and systematic reviews have highlighted the significant effects of inspiratory muscle training (IMT) on improving respiratory muscle strength, particularly in enhancing diaphragm weakness in patients undergoing invasive mechanical ventilation (5, 11). IMT has also been shown to increase maximal inspiratory and expiratory pressure, shorten mechanical ventilation duration, and reduce ICU length of stay (12, 13). Moreover, the total weaning time from mechanical ventilation was shorter in both low-to-moderate intensity and high-intensity threshold IMT groups (14). These findings underscore the importance of respiratory muscle training in clinical practice as an effective strategy for reducing the duration of mechanical ventilation and its associated complications (13). However, the majority of IMT studies have been conducted on patients who failed to wean from mechanical ventilation or required mechanical ventilator use for more than 7 days (12, 15). Since inspiratory muscle weakness and diaphragm atrophy can occur soon after patients undergo mechanical ventilation, we hypothesize that patients would benefit from inspiratory muscle training during the subacute stage, once the cause of intubation has been resolved and their vital signs have stabilized.

Additionally, muscle training has been shown to alter biochemical parameters and clinical laboratory test results in healthy individuals. Fragala et al. reported that increased participation in aerobic and strength exercises was associated with higher creatinine levels, percent oxygen saturation, and iron (16). Furthermore, serum creatinine levels are linked to lean body mass and handgrip strength in healthy individuals (17). Hemoglobin is responsible for transporting oxygen to tissues and its serum level is associated with exercise capacity. Albumin is essential for transporting substances in the blood, such as proteins, hormones, and electrolytes which is crucial for maintaining the osmotic balance. Albumin levels have been found to be associated with strenuous exercise. Creatinine is associated with not only renal function, but also muscle mass. The association between these clinical laboratory values and muscle training in critically ill patients has not yet been explored.

The purpose of this study was to determine if IMT significantly facilitates liberation from mechanical ventilation and improves respiratory muscle strength among subacute critically ill adult patients when compared to no IMT among subacute critically ill adult patients. The secondary aim was to investigate the relationship among IMT, respiratory parameters, and clinical laboratory test results.

## 2 Methods

### 2.1 Study design

This single-center randomized controlled clinical trial was conducted in medical and surgical intensive care units (ICUs) at the Zuoying Armed Forces General Hospital in Kaohsiung City, Taiwan. The Institutional Review Board of the Kaohsiung Armed Forces General Hospital approved the study protocol (approval number: KAFGHIRB107-027). All the measurements complied with the ethical standards of the Declaration of Helsinki. The study registered at ClinicalTrials.gov identifier: NCT06611683, on September 25, 2024. This work was reported in line with the Consolidated Standards of Reporting Trials (CONSORT) Guidelines.

Subjects admitted to the adult ICU were screened daily by a study investigator (S-J, W) who reviewed the bed occupancy in each ICU to identify potential study participants. Electronic medical records were reviewed for inclusion and exclusion criteria. Eligible participants were adult human subjects aged ≥18 years who required invasive mechanical ventilation for >2 days in an ICU. Subjects were excluded from the study if they met any of the following criteria: hemodynamic instability (heart rate > 120 beats/min, unstable blood pressure, vasopressor infusion), inadequate oxygenation (PEEP >8 cmH2O, FiO2 > 50%), body temperature > 38.5°C, sepsis, use of sedative infusion, steroid administration, and home ventilator use before ICU admission. The study investigator approached all eligible participants and invited them to participate in the study. All subjects provided informed consent signed by their proxy before enrollment in the study.

### 2.2 Randomization

We used a simple parallel randomization scheme to allocate subjects to one of the two equal (1:1) groups. A computer-generated random numbers generator (Researcher Randomizer website) randomly allocated the study subjects into the IMT or no-IMT groups. Group allocation was performed before the commencement of the study. Each randomly generated number was printed, shuffled, placed, and sealed in a sequentially numbered opaque envelope by a person not-affiliated with the study. To ensure concealment, the allocation was hidden until the envelope was opened. Study group allocation was concealed from the study investigators, subjects, and all healthcare providers until written informed consent was obtained. At this point, the study investigator was given a sequentially numbered and sealed envelope to open and reveal the group assignments. A double-blind study was not feasible due to the distinct IMT device appearance. Blinding was only applied to the individuals responsible for statistical analysis and outcome preparation.

### 2.3 Inspiratory muscle training

Training was performed by registered respiratory therapists in ICUs. Airway secretions were removed by tracheal suction, and the artificial airway cuff was inflated to prevent potential air leakage before IMT was initiated. Subjects were positioned supine in the bed with the head-of-bed elevated to 60-degrees. They were then removed from the ventilator, and an IMT device was connected to the artificial airway. The threshold IMT device (Galmed Corp., Taiwan) used a starting resistance set to 30% maximum inspiratory pressure (MIP). If the subject tolerated IMT in the previous session, the resistance was increased by 5–10%, up to a maximum of 50% of the MIP. The subjects were instructed to perform fast and forceful inspirations against added inspiratory resistance while being coached by a study investigator to ensure full inspiration and expiration during each breath cycle. Each session consisted of three breaths per set, followed by a one-minute break during which the ventilator was reconnected. Ten sets (totaling 30 breaths) were completed per IMT session, conducted twice daily over five consecutive days, followed by a two-day rest period. This regimen was continued for three consecutive weeks or until the subject no longer required ventilator support. The inhalation port of the IMT device was occluded in subjects who were unable to follow IMT instructions. This action stimulates a strong neural output from the brainstem, enhancing diaphragm myofiber contraction, leading to deeper breathing. Vital signs and subject tolerance of IMT were monitored closely during therapy sessions by performance of regular assessments and solicitation of subject feedback. IMT was discontinued if the subject experienced a respiratory rate > 40 breaths/min, heart rate increase >20 beats/min, systolic blood pressure increase >20 mmHg, SpO2 < 90%, multifocal premature ventricular contraction, cold sweating, agitation, subjective discomfort, or change in consciousness. IMT was terminated if the subject experienced life-threatening arrhythmia twice during the IMT session.

### 2.4 Outcome measures

Baseline demographic and clinical characteristics, including age, sex, acute physiology and chronic health evaluation score, Glasgow Comma score, primary diagnosis, intensive care unit, artificial airway type, ventilator settings, mechanical ventilation duration, and ICU length of stay were collected from each participant’s electronic medical records. The primary outcome measure was the number of days until liberation from mechanical ventilation among subjects who received IMT (IMT group) compared to subjects who did not receive IMT (no-IMT group).

The secondary outcomes of interest were respiratory muscle strength and biomarker level. Measures of respiratory muscle strength included the maximum inspiratory pressure (MIP), maximum expiratory pressure (MEP), peak inspiratory flow, and peak expiratory flow. Respiratory measurements were performed by a designated respiratory therapist. MIP was measured by having each subject exert maximum inspiratory force against a pressure gauge (Galmed Corp, Taiwan) for three consecutive breaths. The highest value obtained from three consecutive maximum inspiratory efforts was recorded. Similarly, peak inspiratory flow was measured using a respiratory mechanics monitor (VENTCheck, Novametrix Medical Systems Inc., Pennsylvania, U.S.) during three forceful inspirations, with the highest of the three peak inspiratory flow measurements being recorded. During forceful expirations, the MEP and peak expiratory flow were measured using the same method. The tidal volume was measured with a respirometer (Haloscale Wright Respirometer, Ferraris Development & Engineering Co., London) during 1 min of quiet breathing, and the respiratory rate was recorded simultaneously. Additionally, the rapid shallow breathing index (RSBI), which is the threshold for determining the likelihood of successful liberation from invasive mechanical ventilation, was recorded. The RSBI was calculated by dividing the respiratory rate by the tidal volume (L). MIP was measured before IMT intervention for threshold resistance adjustment and daily for the no-IMT group during study enrollment or until the subject was successfully liberated from mechanical ventilation within 48 consecutive hours. Additionally, blood samples were obtained to measure and examine biomarkers, including hemoglobin, albumin, and creatinine levels, to determine their relationship with IMT intervention and outcomes.

### 2.5 Statistical analysis

*A priori* power analysis was performed to determine the study sample size before commencement of the study. A review of the literature showed that tracheotomized subjects who received IMT during mechanical ventilation in the ICU experienced seven fewer days on the ventilator than patients who did not receive IMT (18). With alpha set at 0.05, and beta set at 0.2, we estimated that 30 subjects per group would be necessary to power our study to 80%.

Nonparametric statistical analyses were performed because of the small sample size. Continuous variables with non-normal distribution are reported as median (interquartile range), and the Mann–Whitney U test was used for group comparisons. For categorical variables, data were reported as frequency counts and percentages. The frequency count of participants in each category with normal approximation was compared using the chi-square of independence test or Fisher’s exact test if the expected frequency count of a category was less than 5, as appropriate. The relationship between muscle strength and biomarkers was investigated using Spearman’s rho test. All *p*-values were two-tailed, and values less than 0.05 were considered statistically significant. Data were analyzed using SPSS 26 for Windows (IBM, Armonk, New, USA).

## 3 Results

**Figure 1:**
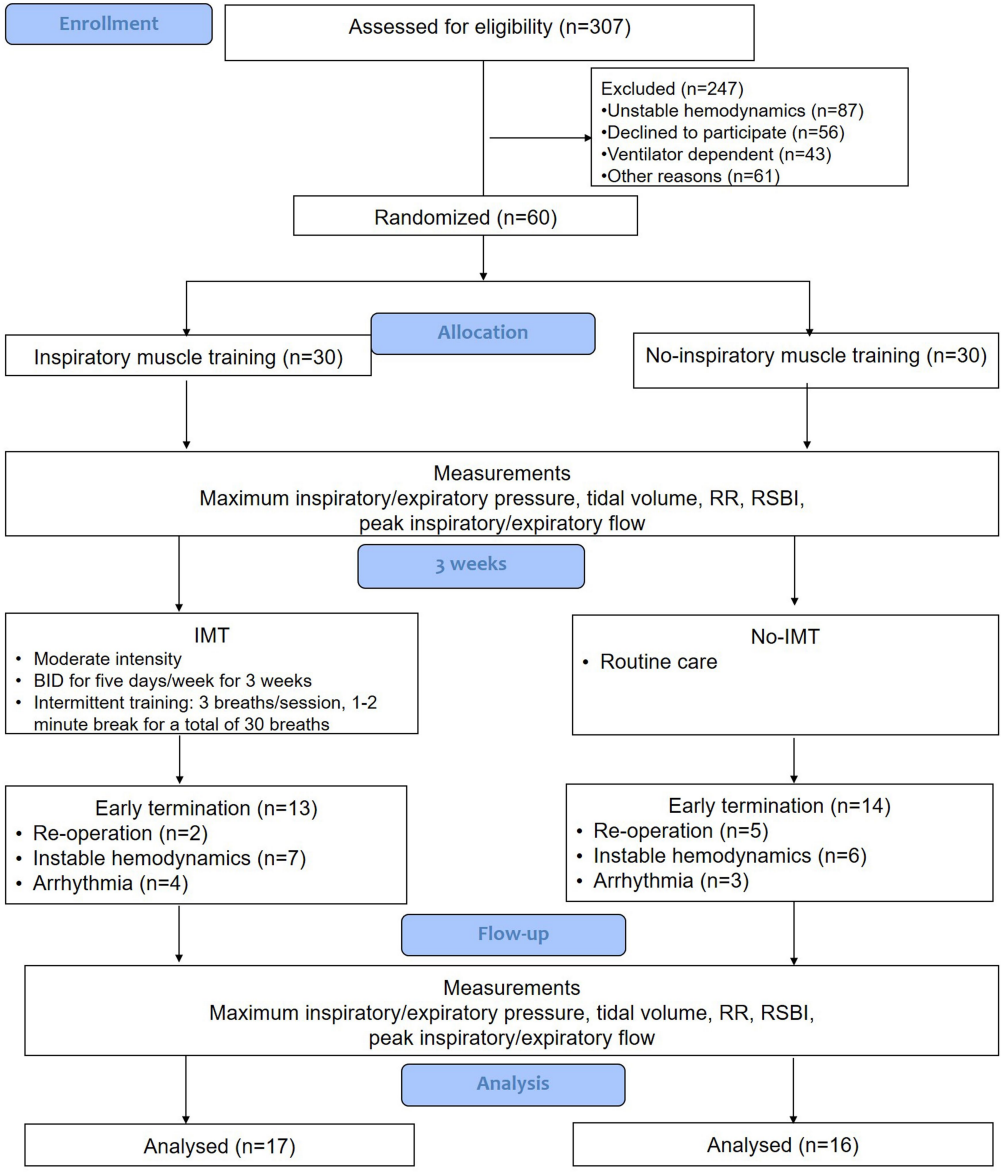
Design and flow of subjects through the trial.

From July 2019 to December 2020, 307 patients who required invasive mechanical ventilation in our medical and surgical ICUs were screened for eligibility. The trial was completed as planned upon reaching a predetermined sample size. Of the 307 screened subjects were excluded from the study enrollment. The most common reason for exclusion was unstable hemodynamics (*n* = 87 of 247). Sixty subjects who met the study inclusion criteria were equally randomized and allocated to either the IMT group (*n* = 30) or the no-IMT group (*n* = 30). Thirteen of the 30 subjects (43%) in the IMT group and 14 of the 30 subjects (47%) in the no-IMT group experienced early study termination (*p* = 0.79). The most common reason for early study termination in both groups was unstable hemodynamics (*p* = 0.57). Seventeen subjects in the IMT group and 16 subjects in the no-IMT group were included in the final data analysis (Figure 1).

The baseline demographic and clinical characteristics of the patients are summarized in Table 1. There were no significant differences between the two groups in terms of age, sex, diagnosis, or baseline clinical variables. The median age was 74 years, and males represented 52% (*n* = 17 of 33) of the study population (*p* = 0.73). The majority (67%) of subjects had a primary diagnosis of a respiratory system disorder (*n* = 14 of 33) or neurological or neurosurgical disorders (*n* = 8 of 33). The majority (84.5%) of subjects received an artificial airway with an endotracheal tube. While 61 % of the subjects were ventilated with Pressure Control mode at the time of study enrollment, when compared to the synchronized intermittent mandatory ventilation + pressure support and pressure support ventilation modes, the difference was not significant (*p* = 0.32).

**Table 1:** Subject demographic and clinical characteristics.

Parameters	IMT (*n* = 17)	No-IMT (*n* = 16)	*p*- value
Age (yr)	74 (69–78)	74 (71–80)	0.30^a^
APACHE II Score	23.5 (19–27)	21 (16–26)	0.24^b^
Glosgow Coma Score	8 (6–9)	9 (7–11)	0.36^b^
Sex: Male, *n* (%)	8 (47.1)	9 (56.3)	0.59^b^
Intensive care unit			
Medical, *n* (%)	13 (76.5)	10 (62.5)	0.38^b^
Surgical, *n* (%)	4 (23.5)	6 (37.5)	
Diagnosis			0.41^b^
Respiratory system disorders, *n* (%)	8 (47.1)	6 (37.6)	
Neurological and Neurosurgical Disorders, *n* (%)	2 (11.8)	6 (37.6)	
Trauma and critical care, *n* (%)	5 (29.5)	2 (12.6)	
Cardiac disorder, *n* (%)	2 (11.8)	2 (12.6)	
Artificial airway type			0.58^b^
Endotracheal tube, *n* (%)	15 (88.2)	13 (81.2)	
Tracheostomy tube, *n* (%)	2 (11.8)	3 (18.8)	
Ventilator setting			0.32^b^
PCV, *n* (%)	10 (58.8)	10 (62.5)	
SIMV+PSV, *n* (%)	3 (17.6)	5 (31.5)	
PSV, *n* (%)	4 (23.5)	1 (6.3)	
PEEP (cmH_2_O)	5	5	> 0.99^a^
FiO_2_ (%)	33 (30–37)	35 (35–40)	0.45^a^

Data are expressed as medians (interquartile ranges). *p* < 0.05 is considered significant. ^a^statistical analysis by Mann–Whitney U test; ^b^statistical analysis by chi-square test.

IMT, inspiratory muscle training; APACHE, Acute Physiology and Chronic Health Evaluation; PCV, pressure control ventilation; SIMV+ PSV, synchronized intermittent mandatory ventilation + pressure support ventilation; PSV, pressure support ventilation; PEEP, positive end-expiratory pressure; FiO2, fraction of inspired oxygen.

Table 2 compares the primary outcomes between the two groups, which were similar at baseline when the subjects were enrolled in the study. Subjects in the IMT group experienced 4.5 fewer days of mechanical ventilation (13 [8–16] days) than those in the no-IMT group (17.5 [9.7–24] days; *p* = 0.038); however, the ICU duration was similar between groups (IMT group 20 [13–30] vs. no-IMT group (20 [17–25]), *p* = 0.92) Subjects who received IMT experienced 4.5 fewer days on mechanical ventilation than those who did not receive IMT (*p* = 0.04). The ICU duration was similar between the two groups. The number of subjects passing the critical point (RSBI <105 breaths/min/L) was significantly greater in the IMT group (*n* = 11) than in the no-IMT group (*n* = 5) at the time of mechanical ventilation liberation (*p* = 0.01).

**Table 2:** Comparisons of outcomes between two groups.

Parameters	IMT (*n* = 17)	No-IMT (*n* = 16)	*p*- value
Mechanical ventilation duration (day)	13 (8–16)	17.5 (9.7–24)	0.038^a^
ICU length of stay (day)	20 (17–25)	20 (13–30)	0.92^a^
RSBI <105 (*n*, %)			
Baseline	2 (11.7%)	4 (25%)	0.58^b^
At the time of MV liberation	11 (64.7%)	5 (31.2%)	0.01^b^

Data are expressed as medians (interquartile ranges). *p* < 0.05 is considered significant. ^a^statistical analysis by Mann–Whitney U test; ^b^statistical analysis by chi-square test.

RSBI: rapid shallow breathing index; MV: mechanical ventilation.

Table 3 compares the muscle strength and biomarkers between groups. The IMT group experienced a significant decrease in RSBI and evidence of significantly improved respiratory muscle strength at the time of MV liberation when compared with baseline measurements. The RSBI results showed a significant reduction in the IMT group (88 breaths/min/L) compared with the no-IMT group (132 breaths/min/L, *p* = 0.04). Additionally, more subjects in the IMT group (*n* = 11) passed the RSBI critical value of <105 breaths/min/L when compared to the no-IMT group (*n* = 5) (Table 2). The secondary outcome of this study was respiratory muscle strength. Subjects who received IMT showed significant increases in MIP (*p* < 0.01), MEP (*p* = 0.03), and peak expiratory flow rate (*p* < 0.01).

**Table 3:** Comparisons of respiratory strength and biomarkers between groups.

	Baseline			At time of MV liberation		
	IMT (*n* = 17)	No-IMT (*n* = 16)	*p*-value	IMT (*n* = 17)	No-IMT (*n* = 16)	*p*-value
RSBI (breath/min/L)	153 (119–181)	125 (102–191)	0.43	88 (65–114)	132 (98–177)	0.042
MIP (cmH_2_O)	26 (18–36)	20 (12.7–24)	0.15	30 (27–36)	22 (13–26)	<0.01
MEP (cmH_2_O)	18 (14.3–29)	20 (16–26)	0.73	28 (21–37)	25 (14–28)	0.03
PIFR (L/min)	24 (18–30)	27 (18–31)	0.55	30 (27–40)	27 (19–31)	0.07
PEFR (L/min)	27 (25–33)	30 (26–31)	0.46	34 (31–37)	29 (21–34)	0.01
Hemoglobin (mg/dL)	10.1 (8.8–11.5)	9.8 (8.8–10.5)	0.45	10.4 (9.3–11)	10.2 (9.7–106)	0.95
Albumin (mg/dL)	3.1 (2.8–3.2)	3.0 (2.8–3.2)	0.83	3.1 (2.6–3.5)	2.9 (2.9–3.2)	0.89
Creatinine (mg/dL)	1.1 (0.8–2.4)	1.2 (0.9–2.5)	0.53	1.2 (0.6–2.4)	0.8 (0.58–1.9)	0.38

Data are expressed as median (interquartile range); ^a^ statistical analysis was performed by Mann–Whitney U test.

IMT, inspiratory muscle training; MIP, Maximum inspiratory pressure; MEP, Maximum expiratory pressure; PIFR, peak inspiratory flow rate; PEFR, peak expiratory flow rate; RSBI, rapid shallow breathing index.

Table 4 demonstrates the changes in measurements between baseline and at the time of MV liberation for the two groups. All changes in respiratory parameters significantly improved, including the RSBI, MIP, MEP, peak inspiratory flow rate, and peak expiratory flow rate. Notably, a significant change in creatinine level was detected between the groups (*p* < 0.01). The variation in creatinine level was demonstrated by a positive moderate correlation with IMT (r_s_ = 0.54, *p* = 0.01).

**Table 4:** Respiratory and biomarker changes from baseline to three-week point.

	IMT (*n* = 17)	No-IMT (*n* = 16)	MD (95% CI)	*p*-value
RSBI %	−35.72 ((−66.94)–0.71)	−9.37 ((−23.2)–31.0)	14.4 (8.8–67.9)	0.01
MIP %	29.41 (7.14–50.0)	9.17 (2.78–21.25)	−25.6 ((−54.2)–3.0)	0.08
MEP %	16.67 (9.09–58.33)	2.08 ((−18.09)–25.55)	−44.6 ((−83.5)–(−5.7))	0.03
PIFR %	36.84 (5.88–78.79)	2.5 ((−21.92)–14.06)	−39.8 ((−68.3)–(−11.3))	0.01
PEFR %	21.21 (9.09–40.0)	−4.95 ((−41.8)–5.11)	−37.1 ((−69.6)–(−4.5))	0.03
Hemoglobin (mg/dL)	0.0 ((−0.9)-0.9)	0.05 ((−0.8)-1.45)	0.54 ((−0.79)–1.88)	0.42
Albumin (mg/dL)	0.0 (0–0.3)	0.9(0.1–(−0.4))	0.15 ((−0.51)–0.09)	0.15
Creatinine (mg/dL)	0.31 (0.93)	−0.72 (0.92)	−1.03 (−1.68–(−0.37))	<0.01

%: change % of the value from the baseline. Data are expressed as medians (interquartile range). *p* < 0.05 were considered significant. Statistical analysis performed by Mann–Whitney U test.

IMT, inspiratory muscle training; MIP, Maximum inspiratory pressure; MEP, Maximum expiratory pressure; PIFR, peak inspiratory flow rate; PEFR, peak expiratory flow rate; RSBI, rapid shallow breathing index (breaths/min/L); MD, mean difference.

## 4 Discussion

Loss of respiratory muscle strength occurs in most critically ill patients and is directly associated with prolonged mechanical ventilation, ICU stay, and hospitalization duration. Our study identified clinical benefits experienced by subacute critically ill adult subjects who received IMT during mechanical ventilation. Our findings indicate that initiating IMT early, once the patient is clinically stable, significantly shortens the duration of mechanical ventilation. This early intervention also enhances respiratory muscle strength and promotes spontaneous breathing, as demonstrated by reduced RSBI and improved respiratory mechanics during the subacute phase. Our data also revealed a moderate correlation between IMT and creatinine levels, potentially indicating increased muscle mass.

### 4.1 Enhanced breathing patterns and facilitation of ventilator weaning in subacute stage

The optimal goal of IMT in critically ill patients is to improve respiratory muscle strength, thereby enhancing respiratory efficiency and facilitating liberation from mechanical ventilation support. Previous studies have used MIP and MEP as the primary outcomes to evaluate the effectiveness of IMT in patients receiving mechanical ventilation (19, 20). Improvement in MIP or MEP measurements reflects enhanced strength of the respiratory muscles during forceful inspiration or expiration, respectively. MIP and MEP serve as critical indicators of effective coughing ability, a vital function for airway secretion clearance, during ventilator weaning (21, 22). Successful ventilator weaning necessitates adequate ventilation capacity, encompassing both strength and endurance.

The primary goal of patient care at the subacute stage of critical illness is liberation from mechanical ventilation. Generally, the RSBI is a clinical predictor used to inform about patient readiness and the likelihood of remaining liberated from mechanical ventilation. A higher RSBI suggests that the patient is breathing rapidly or has a small tidal volume. Although the specificity of the RSBI has been debated, a cutoff value of greater than 105 has been established to predict a higher likelihood of ventilator removal failure (11, 23). Previous studies have shown that a high RSBI indicates an inadequate ventilation pattern, leading to respiratory muscle weakness or an increased ventilation load (23). We coupled routine care with IMT at the subacute critically ill stage as soon as the patients exhibited hemodynamic stability and a supplemental oxygen concentration requirement of less than 50%. Our results demonstrated that IMT improved respiratory muscle strength, endurance, and breathing patterns. Consequently, the duration of mechanical ventilation was significantly reduced by 6 days when IMT was added to routine care.

While the cost of medical care is on the rise, a systematic study estimated that a single ventilated ICU day costs €1,654 in France, with mechanical ventilation accounting for approximately 50% of the daily cost (24). Our study demonstrated a 4.5-day reduction in duration of mechanical ventilation, potentially resulting in cost savings of at least €3,600. Moreover, early extubation helps prevent additional costs associated with mechanical ventilation, such as ventilator-associated pneumonia, ventilator-induced lung injury, and complications resulting from upper airway trauma and artificial airway securing device pressure related skin ulcer injury.

### 4.2 Intermittent moderate-intensity training protocol and duration of training

The training duration and methods employed in previous studies have exhibited discrepancies. Inspiratory muscle training is used in some intensive care units; however, its effectiveness remains uncertain (6). Given that patients were just past the acute phase of critical illness, we designed the IMT with low-to-moderate intensity threshold resistance to prevent intolerance and overuse of the inspiratory muscles. A systematic review of low-to-medium vs. high intensity revealed that the high-intensity threshold IMT groups in critically ill patients concluded that both intensities achieved statistically significant MIP improvement (14). Additionally, low-to-moderate-intensity IMT reduced the duration of mechanical ventilation. In contrast to our study, a systematic review found that RSBI results favored high-intensity IMT over low-intensity IMT.

Previous studies have also shown a wide variation in the duration of IMT interventions, ranging from 3 days to 6 weeks, for patients with diverse medical conditions (11). Bissett et al. conducted a study incorporating 2 weeks of moderate-intensity IMT in patients receiving mechanical ventilation, which demonstrated significant improvements in inspiratory strength and quality of life (19). Their subsequent analysis found that patients who benefited the most from IMT were those with moderate inspiratory muscle weakness (MIP >28 cmH2O) within 48 h of ventilatory weaning (18). Similarly, in this three-week moderate-intensity IMT study, our subjects in the IMT group, with a median MIP of 26 cmH2O, showed significant improvement in respiratory muscle strength and respiratory pattern parameters. Our study demonstrated a significantly shorter duration of mechanical ventilation which likely resulted from the addition of IMT intervention to regular standard of care.

### 4.3 Correlation between creatinine level to IMT

Our results demonstrated significant differences in post-IMT creatinine level changes between subjects who received IMT when compared to subjects who did not receive IMT. We identified a moderate correlation between creatinine level and improvement in respiratory muscle strength. To our knowledge, this study is the first to observe a relationship between creatinine levels and IMT outcomes in adult mechanically ventilated subjects. Muscle weakness is a major concern for critically ill patients who require mechanical ventilation because they are often sedated and underfed during the acute stage of care. Serum creatinine level reflects both creatinine production and elimination, making it a standard clinical biomarker that critical care clinicians use to monitor renal function trends. Creatinine production primarily arises from phosphocreatine metabolism in the skeletal muscle and is correlated with muscle mass (25). Serum creatinine could indicate muscle mass and it is affected by altered nutritional status and skeletal muscle wasting. Low serum creatinine levels are indicative of low muscle mass, malnutrition, fluid overload, and advanced liver diseases (24). A reduction in muscle mass can be determined by a decrease in serum creatinine level. Our study revealed a significant reduction in creatinine level among subjects who did not receive IMT (−0.72 mg/dL) whereas creatine level increased slightly among subjects that received IMT (0.31 mg/dL). The increased creatinine level in the IMT group may indicate maintained inspiratory and diaphragm muscle mass, as opposed to the possibility of muscle wasting in the no-IMT group. Previous studies have found that diaphragmatic atrophy occurs more frequently in patients with septic shock than in those on mechanical ventilation (26, 27). The IMT intervention has been shown to increase diaphragm thickness and improve peripheral muscle strength, peripheral muscle thickness, diaphragm muscle thickness, and diaphragm muscle mobility (28). The increased creatinine level observed in our study may be attributed to diaphragmatic training, which is the largest inspiratory muscle. In contrast, patient hydration status may greatly effect creatinine levels. Future clinical studies are necessary for deeper exploration and external validation of our findings.

### 4.4 Limitations

This study had limitations that must be considered. This single-center RCT was conducted in medical and surgical ICUs, which limits the generalizability of our findings. Recruitment and retention of subjects in the study also presented challenges, reflected by a dropout rate of 43%, when they were in the subacute stage of the illness. Surrogates are often reluctant to consent for subject participation in the study owing to safety concerns. Additionally, patient medical conditions can change rapidly in the subacute stage, which partly explains the high dropout rate observed in both groups and the small sample size included in our final data analysis. To address this issue, we suggest strategies to improve participant retention in future clinical studies. Regularly communicate with study subjects and surrogates to address any study related concerns. Due to the difficult in recruiting subacute critically ill patients, our sample size was small. Larger multicenter clinical trials are warranted in the future. A double-blinded study design was not feasible due to the nature of the IMT device, and as a result, performance bias may have been introduced into our study during IMT training sessions as participants were encouraged to push closer to their limits. However, a blinded approach was implemented during statistical analysis to minimize bias in the interpretation of the results.

## 5 Conclusion

Reducing respiratory muscle strength in critically ill patients often leads to prolonged mechanical ventilation and an extended hospital stay. Our randomized clinical trial demonstrated that IMT facilitates earlier liberation from mechanical ventilation and improves respiratory muscle strength. These findings underscore the potential benefit that IMT may offer as an adjunct to regular standard of care for subacute critically ill adult patients requiring mechanical ventilation. Further research in large clinical trials is needed to explore the relationship between serum creatine levels and respiratory muscle strength. Establishing precise serum creatine and respiratory muscle strength and retention cut-off values that demonstrate clinical benefits could enhance clinical practice and improve patient outcomes.

## Data Availability

The original contributions presented in this study are included in the article, and further inquiries can be directed to the corresponding authors.

## References

[1] Adler D Dupuis-Lozeron E Richard JC Janssens JP Brochard L. Does inspiratory muscle dysfunction predict readmission after intensive care unit discharge? Am J Respir Crit Care Med. (2014) 190:347–50. doi: 10.1164/rccm.201404-0655LE, PMID: 25084264

[2] Kim WY Suh HJ Hong SB Koh Y Lim CM. Diaphragm dysfunction assessed by ultrasonography: influence on weaning from mechanical ventilation. Crit Care Med. (2011) 39:2627–30. doi: 10.1097/CCM.0b013e3182266408, PMID: 21705883

[3] Dres M Dubé BP Mayaux J Delemazure J Reuter D Brochard L. Coexistence and impact of limb muscle and diaphragm weakness at time of liberation from mechanical ventilation in medical intensive care unit patients. Am J Respir Crit Care Med. (2017) 195:57–66. doi: 10.1164/rccm.201602-0367OC, PMID: 27310484

[4] Busl KM Ouyang B Boland TA Pollandt S Temes RE. Prolonged mechanical ventilation is associated with pulmonary complications, increased length of stay, and unfavorable discharge destination among patients with subdural hematoma. J Neurosurg Anesthesiol. (2015) 27:31–6. doi: 10.1097/ANA.0000000000000085, PMID: 24922337

[5] Bissett B Leditschke IA Green M Marzano V Collins S Van Haren F. Inspiratory muscle training for intensive care patients: a multidisciplinary practical guide for clinicians. Aust Crit Care. (2019) 32:249–55. doi: 10.1016/j.aucc.2018.06.001, PMID: 30007823

[6] Bonnevie T Villiot-Danger JC Gravier FE Dupuis J Prieur G Médrinal C. Inspiratory muscle training is used in some intensive care units, but many training methods have uncertain efficacy: a survey of French physiotherapists. J Physiother. (2015) 61:204–9. doi: 10.1016/j.jphys.2015.08.003, PMID: 26365266

[7] Ramli MI Hamzaid NA Engkasan JP Usman J. Respiratory muscle training: a bibliometric analysis of 60 years' multidisciplinary journey. Biomed Eng Online. (2023) 22:50. doi: 10.1186/s12938-023-01103-0, PMID: 37217941 PMC10201471

[8] Ballew SH Zhou L Surapaneni A Grams ME Windham BG Selvin E. A novel creatinine muscle index based on creatinine filtration: associations with frailty and mortality. J Am Soc Nephrol. (2023) 34:495–504. doi: 10.1681/ASN.0000000000000037, PMID: 36735317 PMC10103307

[9] Kim CY Lee JS Kim HD Lee DJ. Short-term effects of respiratory muscle training combined with the abdominal drawing-in maneuver on the decreased pulmonary function of individuals with chronic spinal cord injury: a pilot randomized controlled trial. J Spinal Cord Med. (2017) 40:17–25. doi: 10.1080/10790268.2016.1198576, PMID: 27463071 PMC5376135

[10] Bissett B Gosselink R van Haren FMP. Respiratory muscle rehabilitation in patients with prolonged mechanical ventilation: a targeted approach In: Vincent J-L, editor. Annual update in intensive care and emergency medicine 2020. Cham: Springer International Publishing (2020). 595–609. 10.1186/s13054-020-2783-0 PMC7092518 32204719

[11] Vorona S Sabatini U Al-Maqbali S Bertoni M Dres M Bissett B. Inspiratory muscle rehabilitation in critically ill adults. A systematic review and meta-analysis. Ann Am Thorac Soc. (2018) 15:735–44. doi: 10.1513/AnnalsATS.201712-961OC, PMID: 29584447 PMC6137679

[12] Elkins M Dentice R. Inspiratory muscle training facilitates weaning from mechanical ventilation among patients in the intensive care unit: a systematic review. J Physiother. (2015) 61:125–34. doi: 10.1016/j.jphys.2015.05.016, PMID: 26092389

[13] Magalhães PAF Camillo CA Langer D Andrade LB Duarte M Gosselink R. Weaning failure and respiratory muscle function: what has been done and what can be improved? Respir Med. (2018) 134:54–61. doi: 10.1016/j.rmed.2017.11.023, PMID: 29413508

[14] Patsaki I Kouvarakos A Vasileiadis I Koumantakis GA Ischaki E Grammatopoulou E. Low-medium and high-intensity inspiratory muscle training in critically ill patients: a systematic review and meta-analysis. Medicina (Kaunas). (2024) 60:869. doi: 10.3390/medicina60060869, PMID: 38929486 PMC11205434

[15] Martin AD Smith BK Davenport PD Harman E Gonzalez-Rothi RJ Baz M. Inspiratory muscle strength training improves weaning outcome in failure to wean patients: a randomized trial. Crit Care. (2011) 15:R84. doi: 10.1186/cc10081, PMID: 21385346 PMC3219341

[16] Fragala MS Bi C Chaump M Kaufman HW Kroll MH. Associations of aerobic and strength exercise with clinical laboratory test values. PLoS One. (2017) 12:e0180840. doi: 10.1371/journal.pone.0180840, PMID: 29059178 PMC5653181

[17] Bartholomae E Knurick J Johnston CS. Serum creatinine as an indicator of lean body mass in vegetarians and omnivores. Front Nutr. (2022) 9:996541. doi: 10.3389/fnut.2022.996541, PMID: 36185683 PMC9525150

[18] Tonella RM Ratti L Delazari LEB Junior CF Da Silva PL Herran A. Inspiratory muscle training in the intensive care unit: a new perspective. J Clin Med Res. (2017) 9:929–34. doi: 10.14740/jocmr3169w, PMID: 29038671 PMC5633094

[19] Bissett BMP Wang JM Neeman TP Leditschke IM Boots RP Paratz JP. Which ICU patients benefit most from inspiratory muscle training? Retrospective analysis of a randomized trial. Physiother Theory Pract. (2020) 36:1316–21. doi: 10.1080/09593985.2019.1571144, PMID: 30739584

[20] Bissett BM Leditschke IA Neeman T Boots R Paratz J. Inspiratory muscle training to enhance recovery from mechanical ventilation: a randomised trial. Thorax. (2016) 71:812–9. doi: 10.1136/thoraxjnl-2016-208279, PMID: 27257003 PMC5013088

[21] Postma K Vlemmix LY Haisma JA De Groot S Sluis TA Stam HJ. Longitudinal association between respiratory muscle strength and cough capacity in persons with spinal cord injury: an explorative analysis of data from a randomized controlled trial. J Rehabil Med. (2015) 47:722. doi: 10.2340/16501977-1986 26074331

[22] MacIntyre NR. Respiratory mechanics in the patient who is weaning from the ventilator. Respir Care. (2005) 50:275. PMID: 15691396

[23] Roberts KJ Goodfellow LT Battey-Muse CM Hoerr CA Carreon ML Sorg ME. AARC clinical practice guideline: spontaneous breathing trials for liberation from adult mechanical ventilation. Respir Care. (2024) 69:891–901. doi: 10.4187/respcare.11735, PMID: 38443142 PMC11285503

[24] Kaier K Heister T Motschall E Hehn P Bluhmki T Wolkewitz M. Impact of mechanical ventilation on the daily costs of ICU care: a systematic review and meta-regression. Epidemiol Infect. (2019) 147:e314. doi: 10.1017/S0950268819001900, PMID: 31802726 PMC7003623

[25] Barreto EF Poyant JO Coville HH Dierkhising RA Kennedy CC Gajic O. Validation of the sarcopenia index to assess muscle mass in the critically ill: a novel application of kidney function markers. Clin Nnut. (2019) 38:1362–7. doi: 10.1016/j.clnu.2018.05.031, PMID: 29921462

[26] Vivier E Roussey A Doroszewski F Rosselli S Pommier C Carteaux G. Atrophy of diaphragm and pectoral muscles in critically ill patients. Anesthesiology. (2019) 131:569–79. doi: 10.1097/ALN.0000000000002737, PMID: 31094757

[27] Dot I Pérez-Terán P Francés A Díaz Y Vilà-Vilardell C Salazar-Degracia A. Association between histological diaphragm atrophy and ultrasound diaphragm expiratory thickness in ventilated patients. J Intensive Care. (2022) 10:40. doi: 10.1186/s40560-022-00632-5, PMID: 35986366 PMC9392308

[28] Sari F Oskay D Tufan A. The effect of respiratory muscle training on respiratory muscle strength, diaphragm thickness/mobility, and exercise capacity in patients with systemic lupus erythematosus and associated shrinking lung syndrome. Lupus. (2024) 33:289–92. doi: 10.1177/09612033241226755, PMID: 38194712

[29] De Rosa S Greco M Rauseo M Annetta MG. The good, the bad, and the serum creatinine: exploring the effect of muscle mass and nutrition. Blood Purif. (2023) 52:775–85. doi: 10.1159/000533173, PMID: 37742621 PMC10623400

